# A correlation-based network for biomarker discovery in obesity with metabolic syndrome

**DOI:** 10.1186/s12859-019-3064-2

**Published:** 2019-12-10

**Authors:** Pin-Yen Chen, Allan W. Cripps, Nicholas P. West, Amanda J. Cox, Ping Zhang

**Affiliations:** 1 Menzies Health Institute Queensland, Griffith University, Gold Coast, Australia; 2 School of Medical Science, Griffith University, Gold Coast, Australia; 3 School of Medicine, Griffith University, Gold Coast, Australia

**Keywords:** Network analysis, Obesity, Metabolic syndrome, Inflammation, Immune system, Gut microbiome, Multidimensional data, Multivariate analysis

## Abstract

**Background:**

Obesity is associated with chronic activation of the immune system and an altered gut microbiome, leading to increased risk of chronic disease development. As yet, no biomarker profile has been found to distinguish individuals at greater risk of obesity-related disease. The aim of this study was to explore a correlation-based network approach to identify existing patterns of immune-microbiome interactions in obesity.

**Results:**

The current study performed correlation-based network analysis on five different datasets obtained from 11 obese with metabolic syndrome (MetS) and 12 healthy weight men. These datasets included: anthropometric measures, metabolic measures, immune cell abundance, serum cytokine concentration, and gut microbial composition. The obese with MetS group had a denser network (total number of edges, *n* = 369) compared to the healthy network (*n* = 299). Within the obese with MetS network, biomarkers from the immune cell abundance group was found to be correlated to biomarkers from all four other datasets. Conversely in the healthy network, immune cell abundance was only correlated with serum cytokine concentration and gut microbial composition. These observations suggest high involvement of immune cells in obese with MetS individuals. There were also three key hubs found among immune cells in the obese with MetS networks involving regulatory T cells, neutrophil and cytotoxic cell abundance. No hubs were present in the healthy network.

**Conclusion:**

These results suggest a more complex interaction of inflammatory markers in obesity, with high connectivity of immune cells in the obese with MetS network compared to the healthy network. Three key hubs were identified in the obese with MetS network, involving Treg, neutrophils and cytotoxic cell abundance. Compared to a t-test, the network approach offered more meaningful results when comparing obese with MetS and healthy weight individuals, demonstrating its superiority in exploratory analysis.

## Background

Obesity is a multifactorial disease that dysregulates many different body systems, including the immune system [1] and the gut microbiota [2], leading to increased risk of chronic diseases, including type 2 diabetes mellitus (T2DM), some cancers, and increased mental health problems [1]. Despite extensive research, no specific biomarker profile is clinically recognised to characterise individuals with a greater risk of developing obesity-related disease [3]. A key reason may be failure to consider the interconnected nature of the immune system, host microbiota and metabolic interactions. Many functional studies have now recognised the need for integrated analysis to overcome the issue of redundancy [4, 5], whereby many biomarkers have similar roles, rendering univariate analysis ineffective. Recent technological advances that allow for multiple biomarker analysis are overcoming the limitations associated with biological complexity to better understand the basis of diseases. However, the interpretation and visualisation of the significant amount of data generated from these methods still poses a challenge [6].

Correlation-based network analysis (CNA) has recently become a popular data-mining method as it allows complexity reduction of multidimensional data while still retaining the majority of information needed for interpretation [6]. CNA provides the means to visualise disease-related perturbations of molecular interactions to provide insight into key underlying mechanisms that drive disease development [7]. In biological network analysis, biomarkers are represented as nodes and the links between them as edges. A number of network properties have been developed to allow interpretation of correlation networks [6], including (a) node degree: the number of other nodes to which a given node is significantly correlated, (b) betweenness centrality: the measure of shortest paths between any two nodes that passes through the node in question, and (c) network density: the ratio of existing edges to the total number of possible edges in a network. Using these properties, researchers can also detect highly connected nodes, also known as hubs. These properties were useful in many obesity studies which used CNA to identify key hubs that differ between obese and healthy weight individuals. Walley et al. used a network approach to compare genes in subcutaneous adipose tissue of obesity-discordant siblings [8]. The study found a third of the transcripts to be differentially expressed between lean and obese siblings, with obesity-associated neuronal growth regulator 1 (NEGR1) acting as a central hub. A later study by Wang et al. [9] also used network analysis to identify significant genes between seven discordant monozygotic twins. From this study, at least eight different hub genes were identified. Both Walley et al. and Wang et al. were able to detect central genes affected by obesity, providing insight into future research looking to target specific biomarkers for obesity treatment. However, these studies are limited by their focus on specific areas of the body. Considering obesity being a multifactorial disease and the functional interdependencies of different systems of the human body, a multi-analyte network should be utilised instead.

Studies examining immune profiles [1] and gut microbial composition [10] in obese individuals have found alterations in favour of pro-inflammatory biomarkers when compared to their lean counterparts. A study by Winer et al. has also found high pro-inflammatory to anti-inflammatory biomarker ratios in obese individuals that exacerbate chronic disease development [11]. However, studies have still struggled to find a profile of biomarkers that distinguish individuals more at risk of obesity-related diseases. Due to the multitude of molecular interactions affected by obesity, a holistic approach is required to identify key biomarkers involved. The aim of this study was to use CNA to compare anthropometric measures, metabolic measures, immune cell abundance, serum cytokine concentrations, and gut microbial composition to identify biomarker profiles that distinguish obese with metabolic syndrome (MetS) from healthy weight individuals.

## Results

The molecular interactions associated with obesity were analysed by comparing networks within obese with MetS and healthy weight individuals through CNA. The characteristics of participants from the two distinct groups are described in Table 1. Significant differences were observed in all the key demographic measures, except for age, between the two groups. By design, the obese with MetS group had values outside the healthy range for variables that constitute the criteria for MetS [12]. Two major markers of inflammation, CRP and ESR, were also compared between the two groups, both of which were higher in the obese with MetS group although the difference was not significant for ESR. As obesity has been described as a state of chronic low-grade inflammation [3], the higher levels of inflammatory markers observed in the obese with MetS group was expected. The findings from the exploratory univariate analysis justified the use of other analytical methods to find possible underlying interactions between inflammatory biomarkers.

**Table 1:** Demographic characteristics and metabolic measures in obese with MetS (*N* = 11) and healthy weight (*N* = 12) males

	Obese with Mets (*n* = 11)	Healthy weight (*n* = 12)	*P*-value*
Demographic variables			
Age (Years)	47.74 ± 8.52	40.98 ± 12.36	0.1
BMI (kg/m2)	35.25 ± 3.57	23.05 ± 1.30	< 0.001
Waist (cm)	177.82 ± 10.31	82.71 ± 5.03	< 0.001
Fat Mass (%)	34.2 ± 2.30	20.48 ± 2.52	< 0.001
Muscle Mass (%)	26.3 ± 2.09	36.27 ± 2.87	< 0.001
Visceral Fat	16.64 ± 3.53	5.75 ± 1.45	< 0.001
Metabolic variables			
MetS	3.55 ± 0.69	0.17 ± 0.39	< 0.001
SBP (mmHg)	144.55 ± 13.37	122 ± 4.78	< 0.001
DBP (mmHg)	96.91 ± 9.98	76.58 ± 6.49	< 0.001
Triglycerides (mmol/L)	2.18 ± 0.50	1.10 ± 0.62	< 0.001
Cholesterol (mmol/L)	5.58 ± 1.01	5.08 ± 1.15	0.24
HDL (mmol/L)	1.13 ± 0.18	1.54 ± 0.34	< 0.001
LDL (mmol/L)	3.46 ± 0.87	3.03 ± 0.88	0.22
HbA1c (%)	5.36 ± 0.43	5.23 ± 0.26	0.42
Glucose (mmol/L)	5.74 ± 0.71	5.20 ± 0.33	0.04
CRP (mg/L)	1.77 ± 0.86	0.95 ± 1.04	0.01
ESR (mm/hr)	6.18 ± 4.62	3.58 ± 0.90	0.27

MetS: scored out of a maximum of five based on presence of five defined metabolic syndrome features

*BMI* Body mass index, *BP* Blood pressure, *MetS* Metabolic syndrome, *HDL* High-density lipoprotein, *LDL* Low-density lipoprotein, *HbA1c* Haemoglobin A1c, *CRP* C-reactive protein, *ESR* Erythrocyte sedimentation rate

*P value is based on an unpaired t-test using log-transformed data

**Fig. 1:**
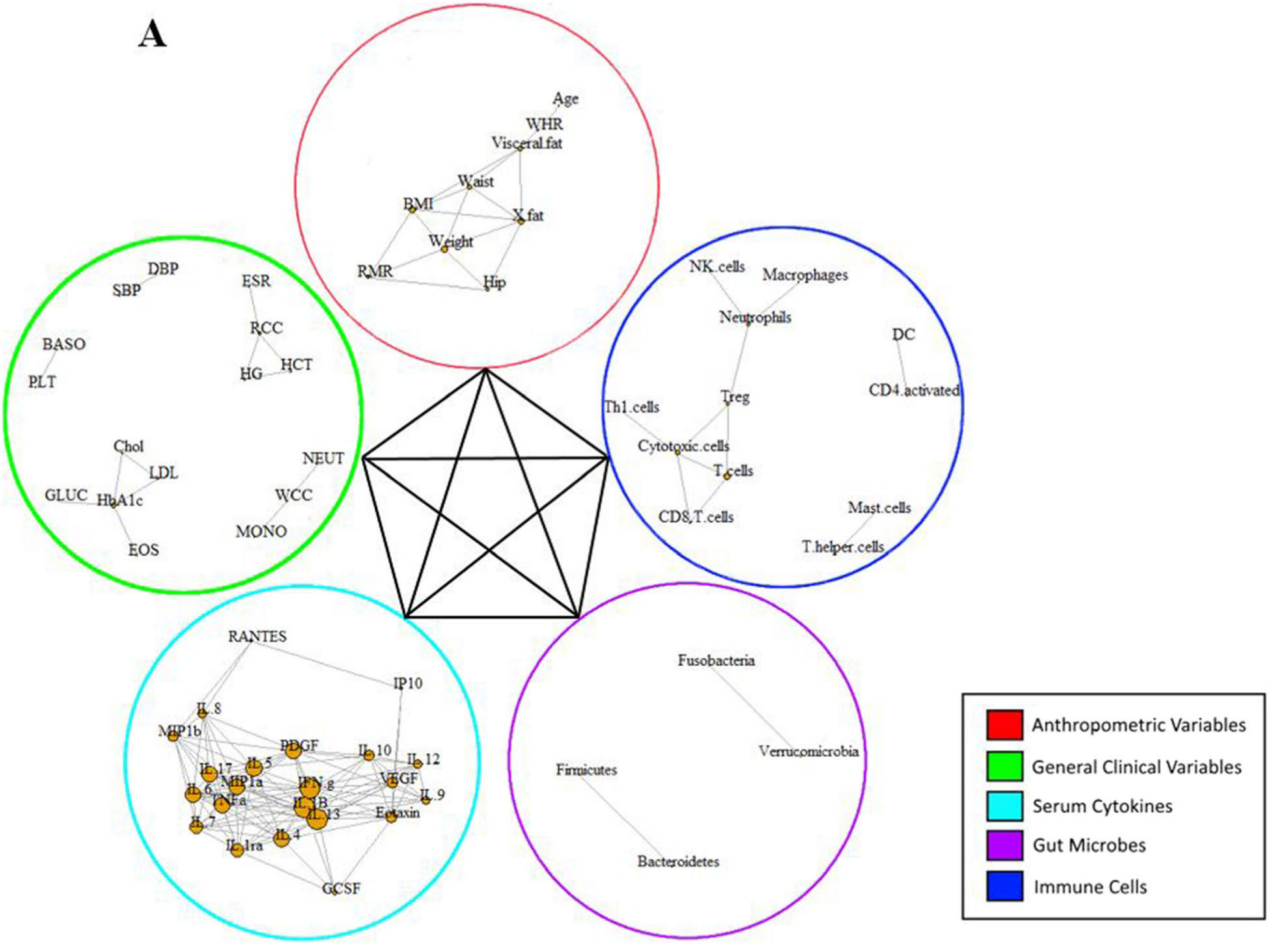
Multi-level CNA constructed for obese with MetS participants. AUSDRISK: Australian type 2 diabetes risk; WHR: waist-hip ratio; BMI: body mass index; X.fat: percentage fat mass; X.musc: percentage muscle mass; RMR: resting metabolic rate; SBP: systolic blood pressure; DBP: diastolic blood pressure; MetS: metabolic syndrome; Chol: cholesterol; LDL: low-density lipoprotein; HDL: high-density lipoprotein; HCT: haematocrit; RCC: red cell count; ESR: erythrocyte sedimentation rate; PLT: platelet; BASO: basophil; CRP: C-reactive protein; LYMPHO: lymphocyte; NK cells: natural killer cells; DC: dendritic cells; Treg: T-regulatory cells; Th1 cells: T-helper 1 cells; VEGF: vascular endothelial growth factor; IL-: interleukin; IP10: interferon gamma-induced protein 10; PDGF: platelet-derived growth factor; IFN.g: interferon gamma; TNFa: tumour necrosis factor alpha; GCSF: granulocyte-colony stimulating factor; MIP1a: macrophage inflammatory protein 1 alpha; MIP1b: macrophage inflammatory protein 1 beta

**Fig. 2:**
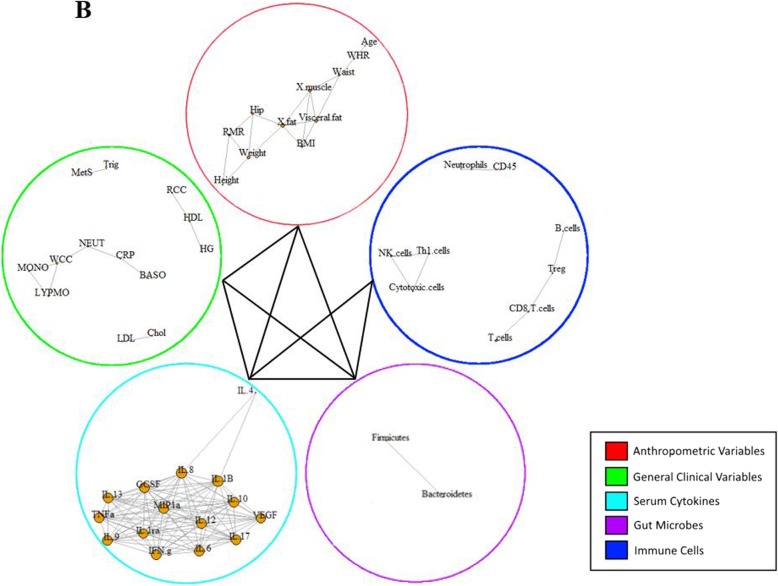
Multi-level CNA constructed for healthy weight (**b**) participants. AUSDRISK: Australian type 2 diabetes risk; WHR: waist-hip ratio; BMI: body mass index; X.fat: percentage fat mass; X.musc: percentage muscle mass; RMR: resting metabolic rate; SBP: systolic blood pressure; DBP: diastolic blood pressure; MetS: metabolic syndrome; Chol: cholesterol; LDL: low-density lipoprotein; HDL: high-density lipoprotein; HCT: haematocrit; RCC: red cell count; ESR: erythrocyte sedimentation rate; PLT: platelet; BASO: basophil; CRP: C-reactive protein; LYMPHO: lymphocyte; NK cells: natural killer cells; DC: dendritic cells; Treg: T-regulatory cells; Th1 cells: T-helper 1 cells; VEGF: vascular endothelial growth factor; IL-: interleukin; IP10: interferon gamma-induced protein 10; PDGF: platelet-derived growth factor; IFN.g: interferon gamma; TNFa: tumour necrosis factor alpha; GCSF: granulocyte-colony stimulating factor; MIP1a: macrophage inflammatory protein 1 alpha; MIP1b: macrophage inflammatory protein 1 beta

A multi-level correlation network was built for the two studied groups (Figs. 1 and 2). In the CNA, each node represented a biomarker that had a strong correlation with another biomarker in the same variable group, denoted by a link between the two nodes based on a Pearson correlation analysis. Correlations between biomarkers from different variable groups was visually represented by a single line connecting the two variable groups involved. The obese with MetS group produced a much denser network compared to the healthy network, with the total number of edges being 369 and 299, respectively. In addition, the obese with MetS network found correlations between biomarkers within each variable group as well as between each of the five variable groups (Fig. 1). Interestingly, immune cells within the healthy network were not found to be correlated with two of the four other biomarker groups: anthropometric measures and metabolic measures (Fig. 2). The high interconnectivity of immune cells in the obese with MetS network compared to the healthy network suggests immune cells to be highly involved in obesity.

The lack of interaction in the healthy network between three variable groups was compared to correlations within the obese with MetS network. While the healthy network found no correlation between biomarkers in the immune cell abundance group and the anthropometric measures group, the obese with MetS network found age to be negatively correlated with Th2 cell abundance (correlation coefficient [ρ] = − 0.74). Furthermore, there was no correlation between biomarkers from the immune cell abundance group with the metabolic measures group. On the other hand, the obese with MetS network found correlations between systolic blood pressure and mast cell abundance (ρ = 0.71), absolute lymphocyte count and macrophage abundance (ρ = − 0.73), absolute lymphocyte count and neutrophil abundance (ρ = − 0.73), and high-density lipoprotein with T-helper cell abundance (ρ = − 0.78).

The increased involvement of immune cells as obesity develops is also supported by the large number of correlations between immune cell biomarkers in the obese with MetS network compared to the healthy network. The obese with MetS network had higher numbers of correlations, higher network density and more biomarkers with high betweenness centrality scores. As betweenness centrality measures the shortest paths between two nodes that passes through the particular node, it signifies how central the biomarker is within a network. A biomarker with a high betweenness centrality score is therefore considered to be a hub in a network that will cause the biggest change on a network if targeted. The obese with MetS network saw 11 correlations between biomarkers, a network density of 0.09 and 3 biomarkers with a high betweenness centrality score of over 0.1 (Table 2). On the other hand, the healthy network only had 7 correlations between biomarkers, a network density of 0.06 and no biomarkers with a high betweenness centrality score (Table 2). The three key hubs of the obese with MetS network stem from the biomarkers: Treg cells (betweenness centrality [BC] = 0.22), neutrophils (BC = 0.20) and cytotoxic cells (BC = 0.15).

**Table 2:** Main properties of the obese with MetS (Fig. 1) and healthy weight networks (Fig. 2)

Network	Total number of edges	Network density	Number of hubs
Obese with MetS	11	0.09	3
Healthy weight	7	0.06	0

Within the immune cell abundance variable group, Treg cell abundance was correlated with neutrophil abundance (ρ = 0.73), cytotoxic cell abundance (ρ = − 0.73) and T cell abundance (ρ = − 0.74); neutrophil abundance was correlated with macrophage abundance (ρ = 0.80) and NK cell abundance (ρ = 0.74), and cytotoxic cell abundance was correlated with Th1 cell abundance (ρ = 0.78), T cell abundance (ρ = 0.77) and CD8^+^ T cell abundance (ρ = 0.74). Additionally, Treg cell abundance was correlated with MIP-1β concentration (ρ = 0.71) from the serum cytokine group while neutrophil abundance was correlated with a number of biomarkers from the gut microbial group, including: *Escherichia*/*Shigella* abundance (ρ = 0.74), *Akkermansia* abundance (ρ = 0.71), *Anaerostipes* abundance (ρ = 0.72), *Blautia* abundance (ρ = 0.78), *Flavonifractor* abundance (ρ = 0.73), and *Holdemania* abundance (ρ = 0.70). These correlations may be considered for intervention studies looking to reduce the prevalence of obesity-related diseases.

An unpaired t-test was also performed on the same dataset (Table 3) and the results were compared to that of the CNA. For the immune cell abundance variable group, the only significant difference found between the healthy weight and obese with MetS groups was in mast cell (*P* = 0.02) and T-helper cell abundance (*P* = 0.04). Mast cell abundance was negatively correlated with T-helper cell abundance, with no correlations with any other immune cells for either of the two biomarkers. Compared to the t-test, the CNA was able to reveal more detailed information on the differences between obese with MetS and healthy weight individuals, demonstrating the importance of using multivariate analysis rather than univariate.

**Table 3:** Immune cell abundance measures in obese with MetS (*N* = 11) and healthy weight (*N* = 12) males

Immune cells	Obese with Mets (*n* = 11)	Healthy weight (*n* = 12)	*P*-value*
Mast cells	3.55 ± 0.68	4.22 ± 0.65	0.02
sNK cells	7.34 ± 0.29	7.26 ± 0.43	0.56
CD8 T cells	6.98 ± 0.53	7.07 ± 0.41	0.64
DC	2.02 ± 0.55	1.7 ± 0.63	0.24
Treg	3.8 ± 0.34	3.89 ± 0.5	0.72
CD45	12.44 ± 0.26	12.33 ± 0.24	0.31
Macrophages	8.89 ± 0.25	8.84 ± 0.23	0.62
T cells	8.88 ± 0.17	8.93 ± 0.18	0.49
Neutrophils	11.01 ± 0.37	10.96 ± 0.33	0.73
Cytotoxic cells	9.34 ± 0.57	9.3 ± 0.51	0.89
Th1 cells	5.49 ± 0.49	5.37 ± 0.66	0.56
Normal mucosa	3.36 ± 0.47	3.25 ± 0.39	0.60
T-helper cells	8.22 ± 0.14	8.32 ± 0.07	0.04
B cells	7.04 ± 0.7	7.12 ± 0.69	0.78
Th2 cells	3.36 ± 0.34	3.9 ± 0.96	0.11
CD4 activated	2.24 ± 0.59	2.1 ± 0.53	0.61

*P value is based on an unpaired t-test using log-transformed data

## Discussion

Many systems of the body have been reported in the literature as being dysregulated in obesity and subsequently increasing the risk of chronic disease development. Due to the complexity of the human body, integrated networks are necessary to better understand the intricate interactions between biomarkers involved in obesity-related diseases. CNA was performed on various datasets obtained from 11 obese men with MetS and 12 healthy weight men. Datasets included were: anthropometric measures, metabolic measures, immune cell abundance, serum cytokine concentrations, and gut microbial composition. Until recently, functional studies in obesity have had conflicting outcomes due to the issue of redundancy and functional interdependencies between biomarkers across different body systems. The aim of this study was to compare the networks constructed for the two studied groups and identify key biomarker interactions that may characterise obesity and related diseases.

When comparing the networks constructed for each group, the obese with MetS group had a denser overall network than the healthy weight group. The differences in the number of correlations suggest the obese with MetS network displayed a more complex connectivity compared to the healthy weight group. The concept of a more complex network confirms the paradigm that obesity is associated with an alteration of multiple parameters across a broad range of biological systems. The interconnected nature of different body systems calls for the need to utilise integrated analytical approaches to deconstruct the complexity of the biological dysregulation in obesity. Through this approach, biomarkers that may be central for investigation in future studies may be identified. The correlation network analysis used in this study supports the use of cluster-based analysis to better understand obesity-related diseases.

In the obese with MetS network, biomarkers of each individual variable group were found to be correlated with other biomarkers from their own group as well as other variable groups. On the other hand, immune cell biomarkers in the healthy weight network were not shown to be correlated with biomarkers from two other variable groups: anthropometric measures and metabolic measures. The contrast between correlations in the obese with MetS and healthy weight networks suggest immune cells to be heavily perturbed in obesity. Both human and animal studies have reported on obesity-related changes in the immune cell abundance and activity which were linked with the development of chronic diseases [13–16]. The similarity in findings between the current study and previous literature suggests CNA to be a reliable analytical method which can be used in studies looking at diseases with complex aetiology.

Further comparisons between the two networks in relation to immune cell abundance revealed more correlations in the obese with MetS network compared to the healthy weight network, with 11 and 7 correlations, respectively. Within the correlations in the obese with MetS network, there were three biomarkers with high betweenness centrality scores. Betweenness centrality is a measure of the number of shortest paths between two other biomarkers that passes through the biomarker in question. A high betweenness centrality score would therefore suggest the biomarker to be the centre of a key hub within the network. The three central biomarkers were: Treg cell abundance, neutrophil abundance and cytotoxic T cell abundance. The correlations found in our study that constitute these hubs have shown positive correlations between pro-inflammatory biomarkers, such as between neutrophils and macrophages, and negative correlations between pro-inflammatory and anti-inflammatory biomarkers, including Treg cells and cytotoxic cells. These correlations are consistent with the findings from earlier studies which have reported a dysregulation in the immune system of obese individuals, resulting in a high pro-inflammatory-to-anti-inflammatory biomarker ratio [11]. All biomarkers have connections with a number of other biomarkers and therefore the recognition of key hubs is crucial in identifying biomarker profiles that characterise obesity-related diseases.

While correlation networks are particularly useful in discovering correlations between biomarkers and key hubs of a system, unpaired t-tests reveal very little in comparison. Performed on the same immune cell abundance data, an unpaired t-test between the obese with MetS and healthy weight group only observed significant differences in mast cell and T-helper cell abundances. Both mast cell and T-helper cell abundances were higher in the healthy weight group. In a study by Liu et al., mast cells contributed to obesity by producing pro-inflammatory cytokines [14]. Therefore, mast cell abundance is expected to be higher in the obese with MetS group, inconsistent with the findings from the current study. Additionally, neither mast cell nor T-helper cell abundance were present in any of the three key hubs found in the obese with MetS network, suggesting the findings from the t-test to be uninformative. The clear distinction between the results of the correlation network and t-test is attributable to the inability of linear causality models to account for the complexity of human body systems.

Using correlation networks, the current study also found many interesting relationships, such as a positive correlation between pro-inflammatory neutrophils and anti-inflammatory Tregs. As obese individuals typically have a high pro-inflammatory-to-anti-inflammatory ratio, this finding was unexpected. A possible explanation for this relationship is suggested in a study by Mishalian et al. who observed the ability of neutrophils to recruit Tregs, exacerbating the impairment of the immune system in disease [17]. Without the use of CNA, a finding that is pertinent in better understanding this multi-factorial disease would be missed in a simple t-test. Relationships between biomarkers, such as neutrophils and Tregs, are important in intervention research which may consider targeting both biomarkers for an exacerbated effect.

Both Treg and neutrophil abundances were also correlated with biomarkers outside of the immune cell abundance variable group. Treg cell abundance was positively correlated with serum MIP-1β concentration, consistent with the findings of Patterson et al., whereby stimulated Tregs produced MIP-1β to assist with T cell migration [18]. Our study also found neutrophils to be associated with a number of gut microbes which is also consistent with earlier studies [19–22]. Neutrophil abundance was positively correlated with gut microbes belonging to neutrophil-associated microbiomes [23]: Firmicutes (*Anaerostipes*, *Blautia*, *Flavonifractor* and *Holdemania*) and Proteobacteria (*Escherichia/Shigella*) phyla. The correlations between biomarkers from different variable groups demonstrates the complexity of interactions between physiological systems and the importance of utilising multi-analyte networks when analysing diseases with complex aetiology.

The differences in results obtained in univariate and multivariate analysis highlights the biggest advantage to using CNA in high-throughput studies. Multivariate analysis allows researchers to consider underlying connections between biomarkers, both within the same or across different variable groups. A simple comparison of biomarker levels between groups does not have the ability to recognise key hubs within a network which may be targeted for future intervention studies. Multivariate analysis has the means to overcome the limitation of redundancy among biomarkers which has limited the ability of functional research to identify key biomarkers in obesity-related disease. Other advantages to using multivariate CNA includes its ease of use and interpretability. The use of correlation networks should therefore be considered for exploratory analysis, rather than unpaired t-test, prior to the use of more complex analytical tools.

The limitations of this study have also been recognised, in particular the small sample size that was used. As a pilot study, the current work was exploratory and utilised high correlation coefficient cut-offs rather than *p*-values to define important results. Another limitation is the small number of molecular markers included in the analysis. While many obesity studies examined markers within adipose tissue, the current study performed analysis on peripheral blood to examine systemic rather than peripheral immune dysregulation. Additionally, the current study did not consider the effects of participant ethnicity in genetic analysis which may result in false positive findings. However, from the known participant ethnicities, 70% were Caucasian, 0.04% were Hispanic and the remaining were unknown. Despite these limitations, the study was still able to gather a multitude of results that supports further research with larger sample sizes and datasets.

## Conclusion

Our study found that obesity with MetS is associated with a more densely connected and therefore complex interaction between inflammatory, gut microbial and metabolism in comparison to that observed in healthy weight individuals. Further analysis revealed immune cells to be highly involved in obesity, with three key hubs in the obese with MetS network that consisted of Treg, neutrophils and cytotoxic cell abundance. The results from the network analysis were much more informative compared to a t-test, suggesting it to be a better choice as an exploratory analytical tool. Our findings demonstrate the need for integrated analysis of multidimensional data to identify specific and multiple interactions between biomarkers that may be targeted for treatment strategies.

## Methods

### Study design and ethics

A correlation-based network analysis was performed on anthropometric measures, metabolic measures, immune gene expression, serum cytokine concentrations and gut microbial composition collected from 12 healthy weight men and 11 obese men with MetS, defined as per the Adult Treatment Panel III criteria [12] (Three or more of the following risk factors: (1) abdominal obesity: ≥30 kg/m^2^ BMI or > 94 cm waist circumference; (2) high blood pressure: ≥130/≥85 mmHg; (3) high triglycerides: ≥1.7 mmol/L; (4) low HDL cholesterol: ≤1 mmol/L; (5) high fasting plasma glucose: ≥6.1 mmol/L or ≥ 6.5% HbA1c). All participants were aged between 18 and 65 years without a history of medical conditions known to affect the immune system, including: cancer, Crohn’s disease, liver disease, and irritable bowel syndrome. Additionally, participants were excluded if they used any immune-modulating medications or supplements, such as: non-steroidal anti-inflammatory drugs (NSAIDs), fish oil and probiotics. Ethics for this study was approved by the Griffith University Human Research Ethics Committee (MED 18.15.HREC) and all participants provided written informed consent prior to their involvement in the study.

### Sample collection and analysis

Fasting blood samples were collected for analysis of metabolic (lipids, glucose, glycated haemoglobin [HbA1c]) and inflammatory (C-reactive protein [CRP], erythrocyte sedimentation rate [ESR], circulating cytokines) measures. In addition, RNA was isolated and analysed using an immune profiling panel of 770 genes (nCounter® PanCancer Immune Profiling Panel, NanoString Technologies, Washington, USA) to estimate the abundance of different immune cells, including mast cells, neutrophils and different T cell subsets. Faecal samples were also collected and microbial compositional sequencing was undertaken via 16S rRNA sequencing and taxonomic classification.

### Correlation-based network analysis

To compare key demographic measures of obese with MetS and healthy weight participants, an unpaired t-test was used and measures were expressed as mean ± standard deviation. Differences in measures were considered significant if the *p*-value was less than 0.05. The dataset was split into five different variable groups: anthropometric measures, metabolic measures, immune cell abundance, serum cytokine concentrations, and gut microbial composition.

**Fig. 3:**
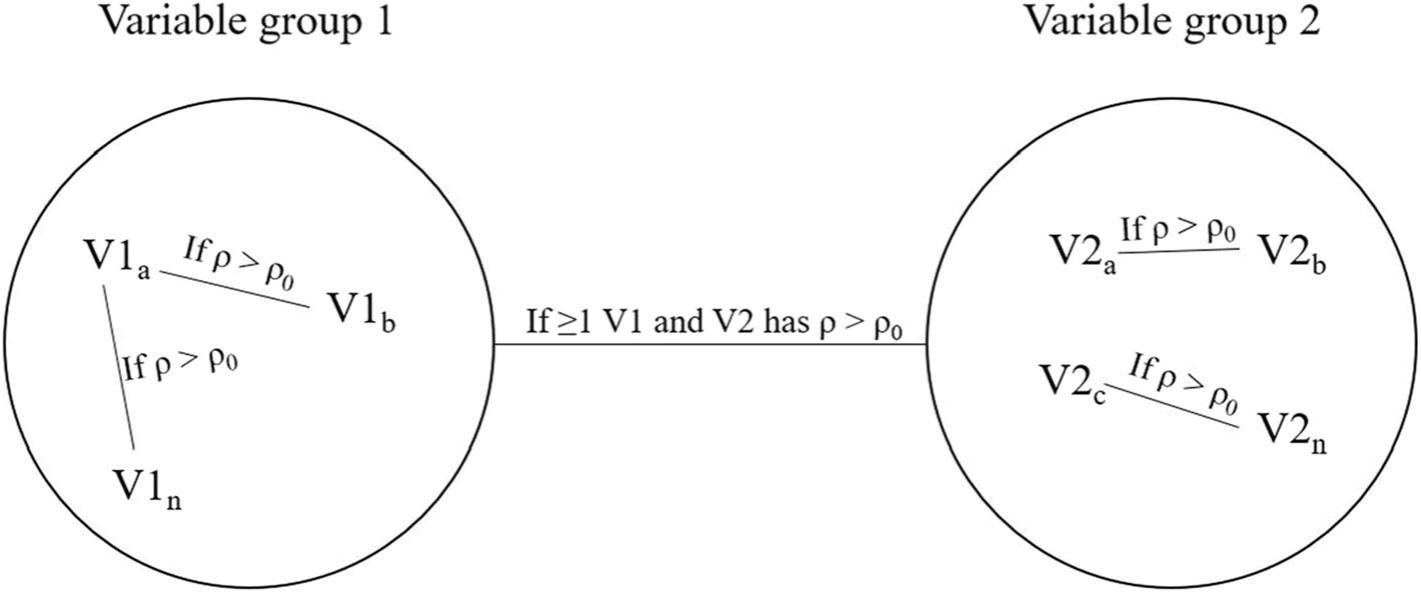
Example of a multi-analyte network constructed in the current study. If two biomarkers within a variable group has a ρ value greater than the initial ρ_0_ threshold specified, the two biomarkers will be connected by a line. Biomarkers without correlations with another biomarker, or with ρ values smaller than ρ_0_, will not appear in the network. If one or more biomarker from one variable group is correlated with one or more biomarker from another variable group with a ρ value greater than ρ_0_, a single line will connect the two variable groups, regardless of the actual number of correlations present

Correlation networks were constructed by firstly calculating the Pearson correlation coefficient (ρ) for each biomarker with all other biomarkers in the five variable groups. A Pearson correlation coefficient threshold was set at | ± 0.7|. Two biomarkers with a correlation coefficient greater than the threshold will be considered as having a strong correlation, visually represented by a link between the two nodes (Fig. 3). Biomarkers that had a strong correlation with another biomarker appeared in the CNA. Strong correlations between biomarkers of different variable groups were indicated by a single line connecting the two variable groups involved, regardless of the total number of correlations found. Nodes of biomarkers without strong correlations with any other biomarker were not included in the CNA. Due to the small sample size, the Pearson correlation coefficient threshold required for a correlation to be considered significant was set very high rather than using a significance level. A complete case correlation analysis was conducted, meaning that biomarkers with missing data were excluded from the network analysis.

All the variables involved in the correlation analysis were continuous variables. Node degree and betweenness centrality was calculated for each node in the correlation network and network density was computed for each variable group. The node degree is the number of strong correlations a particular node has with other biomarkers. Different node sizes in the network visually demonstrate the degree of each node, with a bigger sized node representing a greater node degree. Betweenness centrality scores describe the number of shortest paths between any two biomarkers that passes through the node in question. Nodes with higher betweenness centrality scores are more well-connected within the network and therefore are considered to be drivers of the network. As each variable group has different numbers of nodes, it is difficult to compare betweenness centrality scores across variable groups. Instead, the essentiality of nodes in a network was determined by a high node degree and a high ranking of betweenness centrality score within their respective variable groups. The variable groups from the obese with MetS and healthy networks were compared based on network density, which is the ratio of existing connections to the total number of possible connections within a network. The higher the network density, the more connections there are in the network.

All the statistical analyses and network analyses were carried out with custom R (R Development Core Team, R Foundation for Statistical Computing, Vienna, Austria) scripts. To avoid computational issues that may occur with a large sample size, the code was developed into multiple modules. Each type of analysis, for example Pearson correlation coefficient calculation or network visualisation, had its own module.

## Data Availability

Data are available upon request from the Menzies Health Institute Queensland for researchers who meet the criteria for access to confidential data.

## Abbreviations

BC: Betweenness centrality
BMI: Body mass index
CNA: Correlation-based network analysis
CRP: C-reactive protein
ESR: Erythrocyte sedimentation rate
HbA1c: Haemoglobin A1c
HDL: High-density lipoprotein
MetS: Metabolic syndrome
MIP-1β: Macrophage inflammatory protein 1 beta
NEGR1: Neuronal growth regulator 1
NK: Natural killer
NSAID: Non-steroidal anti-inflammatory drugs
RNA: Ribonucleic acid
rRNA: Ribosomal ribonucleic acid
T2DM: Type 2 diabetes mellitus
